# An invited commentary “Effect of acute normovolemic hemodilution on coronary artery bypass grafting: A systematic review and meta-analysis of 22 randomized trials”

**DOI:** 10.1016/j.amsu.2020.10.056

**Published:** 2020-10-28

**Authors:** Chen Hua Lei, Julius Wedam Atogebania

**Affiliations:** a Department of Surgery, The Second Affiliated Hospital of Hainan Medical University, 570311, Hainan-Haikou, China; b Department of Surgery, The First Affiliated Hospital of Hainan Medical University, 570100, Haikou, China

## 1 Dear editor

Acute normovolemic hemodilution (ANH), a type of surgical intraoperative blood salvage technique used recently more often over homologous blood transfusion during cardiovascular surgery, the technique still remains controversial as postoperative bleeding and transfusion remain a source of morbidity and cost after open heart operations [1].

Shengping Li [1] et al., reported on randomized controlled trials (RCTs) that compared the efficacy of ANH and standard intraoperative care for CABG, the study was performed to enable us better understand the role of acute normovolemic hemodilution (ANH) in a surgical setting with high risk of bleeding, analysis on all randomized controlled trials (RCTs) in the setting of coronary artery bypass grafting (CABG) that compared ANH with usual intraoperative care was conducted via a searched on Cochrane Library, PubMed, EMBASE, Web of Science and CNKI up to April 1, 2020. Two reviewers assessed trial quality and extracted data independently. All statistical analyses were performed using standard statistical procedures provided in Review Manager 5.2. Data for the comparative analysis of ANH versus usual care for CABG were extracted independently by two reviewers, and disagreement was resolved through discussion. The extracted contents, including first authors, published years, country, sample size, surgical type, interventions, baseline hemoglobin and quality score of each study, were displayed using a standardized form. Data collected were input into RevMan 5.2 software for analysis [2].Standard mean difference (SMD) and risk ratio (RR) with its 95% confidence intervals (CIs) were estimated to compare the outcomes of the groups. The primary outcome was to assess the incidence of ANH-related number of allogeneic red blood cell units (ARBCu) transfused. Secondary outcomes included the rate of allogeneic blood transfusion and estimated total blood loss. The study model disclosed that the present meta-analysis indicated that ANH could reduce the number of ARBCu transfused in the CABG surgery setting. In addition, ANH could also reduce the rate of patients transfused with allogeneic blood and estimated total blood loss.

Shengping Li [1] et al., analyzed results were reported on the bases of included studies, study characteristics, and quality assessment, effect of ANH on ARBCu transfused, effect of ANH on the rate of allogeneic blood transfusion, effect of ANH on the rate of estimated total blood loss, long-term follow-up questionnaires, on factors associated with postoperative pain and discomfort. further subgroup analysis were performed in all categories. A total of RCTs including 1688 patients were identified for the present meta-analysis. Of these studies, nineteen were about CABG with on-pump and three with off-pump. Shengping Li [1] et al.*,* pooled results indicating that patients received ANH experienced fewer ARBCu transfusions, with a SMD of −0.60 (95%CI −0.96 to −0.24; P = 0.001). The rate of allogeneic blood transfusion in ANH group was significantly reduced when compared with controls, with a RR of 0.65 (95%CI 0.52 to 0.82; P = 0.0002). In addition, they also experienced less postoperative estimated total blood loss, with a SMD of −0.53 (95%CI −0.88 to −0.17; P = 0.004). In summary de Shengping Li [1] et al., disclosed with evidence that the present meta-analysis show ANH could reduce the number of ARBCu transfused in the CABG surgery setting. In addition, ANH could also reduce the rate of patients transfused with allogeneic blood and estimated total blood loss. Similar reports of advantages of ANH were reported in other studies [3,4] However, no significant difference of estimated total blood loss was found in Jadad scale of 4–5 as subgroup analysis of sample size, significant reduction was observed in sample size < 100 patients, but not sample size ≥ 100 patients. Significant reduction of the rate of estimated total blood loss was found in type of blood replacement with colloids but no crystalloids. Although several limitations have been discussed in this clinical study, other limitations enlightened were, only a restricted number of trials used the same hemodilution procedure and performed volume replacement with the same substance; colloids were extensively used for volume replacement in the ANH group and because they increase the risk of bleeding when compared with crystalloids, they might have worsened the coagulation, resulting in decreased ANH efficacy. In addition, even though there was no difference in the results, significant difference was found with sensitivity analyses. Heterogeneity was observed for the primary and secondary outcomes, suggesting that large, high-quality RCTs are necessary to reach more conclusive results. All these create room for further interesting futuristic studies and researches on effect of acute normovolemic hemodilution on coronary artery bypass graft.

## Sources of funding

No source of funding.

## Ethical Approval

Ethical Approval was issued by the First Affiliated Hospital of Hainan Medical University.

## Research Registration Unique Identifying Number (UIN)

1.Unique Identifying number or registration ID: Not required

## Author contribution

Dr. Chen Hua Lei：Main First Author lead the team in modules, research findings, data organization and analysis, co-assisted with review.

Dr. Julius Wedam Atogebania: Correspondent Author also co-lead the team in writing of scripts, Data analysis, Writing- Original draft preparation, conceptualization , methodology ,software ,assisted with professional protocol guidelines and advancements , follow up on subjects, and other essential matters geared towards a successful completion of the study and review.

## Guarantor

Dr. Julius Wedam Atogebania, MBBS, MD.

## Provenance and peer review

Invited commentary, Editor reviewed.

## Declaration of competing interest

All authors contributed equally;Dr. Chen Hua Lei is the First Author of the article , Corresponding Author is Dr. Julius Wedam Atogebania and others as presented.
